# BUB1B Promotes Ovarian Cancer Cell Proliferation and Metastasis by Activating the Wnt/β‐Catenin Pathway

**DOI:** 10.1002/cam4.71311

**Published:** 2025-10-29

**Authors:** Jing Wang, Xiaoling Su, Nan Lin, Tao Su

**Affiliations:** ^1^ The International Peace Maternity and Child Health Hospital, School of Medicine, Shanghai Jiao Tong University Shanghai China; ^2^ Shanghai Key Laboratory of Embryo Original Diseases Shanghai China; ^3^ Department of Obstetrics and Gynecology Naval Medical Center of PLA Shanghai China

**Keywords:** BUB1B, metastasis, ovarian cancer, proliferation, Wnt/*β*‐catenin pathway

## Abstract

**Background:**

BUB1 mitotic checkpoint serine/threonine kinase B (BUB1B) has been found to participate in cancer progression. Nevertheless, the function and mechanism of BUB1B in ovarian cancer (OC) remain unknown.

**Method:**

Based on datasets GSE14407, GSE18520, and the TCGA combined GTEx database, the differently expressed genes (DEGs) between OC tissues and para‐carcinoma tissues were identified. These DEGs were subjected to protein–protein interaction analysis to obtain hub genes, with BUB1B serving as a candidate for further study. Subsequently, the prognostic value was analyzed. To elucidate the role of BUB1B, knockdown experiments were conducted in vitro and in vivo to assess alterations in malignant behaviors, while *β*‐catenin expression was quantified by qRT‐PCR and western blot. Moreover, the Wnt/*β*‐catenin pathway inhibitor LGK974 was utilized to demonstrate whether the effects of BUB1B are mediated by the Wnt/*β*‐catenin pathway.

**Results:**

High BUB1B expression was observed in OC tissues and cell lines, and it was identified as a hub DEG with prognostic value in OC. Following BUB1B knockdown, cell proliferation, migration, invasion, and tumor growth and metastasis were suppressed in vitro and in vivo. Mechanically, BUB1B knockdown inhibited the expression of *β*‐catenin and inactivated the Wnt/*β*‐catenin pathway. In addition, BUB1B overexpression promoted the malignant behavior of OC cells, which was inhibited by LGK974.

**Conclusion:**

BUB1B is an oncogene whose expression level is negatively correlated with the prognosis of OC patients. Mechanically, BUB1B promotes the progression of OC via the Wnt/*β*‐catenin pathway. Our study offers a potential therapeutic target for OC treatment.

**Abbreviations**
BUB1B: BUB1 mitotic checkpoint serine/threonine kinase B
DEGs: differently expressed genes
EMT: epithelial–mesenchymal transition
HCC: hepatocellular carcinoma
OC: ovarian cancer
SAC: spindle assembly checkpoint

## 1 Introduction

Ovarian cancer (OC) is the second most deadly gynecologic malignancy among women worldwide. In 2020, over 300,000 new cases of ovarian cancer and 200,000 deaths were recorded around the world [1]. At present, surgery and chemotherapy constitute the primary treatments for OC. With the development of targeted therapy, treatment outcomes have been significantly improved. However, fewer than half of OC patients can live longer than 60 months from diagnosis. The high mortality rate is mainly attributed to over 80% of OC patients being diagnosed at an advanced stage, which is often accompanied by severe metastasis [2]. OC patients with metastasis have a poor prognosis despite surgery and often develop drug resistance. Therefore, understanding the molecular mechanisms underlying the progression and metastasis of OC may improve treatment and overall survival.

BUB1 mitotic checkpoint serine/threonine kinase B (BUB1B) is one of the core elements of the spindle assembly checkpoint (SAC), which delays the onset of anaphase and ensures genomic stability by arresting cell division until proper chromosome segregation in mitosis and meiosis [3]. Considering the essential function of BUB1B in mitotic checkpoint signaling and chromosome congression, abnormal BUB1B function results in diseases such as various cancers. High BUB1B expression is associated with poor prognosis and recurrence of multiple types of cancers, including pancreatic [4], prostate cancer [5], liver cancers [6], thyroid carcinoma [7], and OC [8]. BUB1B is linked to malignant cancer traits, such as metastasis [9], proliferation [10], and cell apoptosis [11]. Mechanically, BUB1B promotes malignancy by activating the mTORC1 signaling pathway [12]. In lung adenocarcinoma, BUB1B was found to impair the SAC in mitosis and promote tumor cell proliferation and metastases [10]. In addition, BUB1B promotes cell proliferation and invasion via the JNK‐c‐Jun signaling pathway [13]. However, whether BUB1B plays a role in cancer metastasis and the relevant mechanisms remains unknown.

The Wnt/*β*‐catenin signaling pathway is a well‐characterized oncogenic pathway that plays a pivotal role in ovarian cancer pathogenesis [14]. In the absence of Wnt ligands, *β*‐catenin undergoes phosphorylation and is degraded by a destruction complex composed of AXIN, APC, CK1, GSK3*β*, and *β*TrCP. Wnt ligands bind to cell surface receptors to inactivate the destruction complex. Therefore, β‐catenin could translocate to the nucleus and initiate the transcription of target genes [15]. Notably, frequent mutations in components of this pathway have been reported, resulting in constitutive activation of Wnt/*β*‐catenin signaling [16]. Aberrant activation of the Wnt/*β*‐catenin pathway contributes to the maintenance of cancer stemness, metastasis, chemoresistance, angiogenesis, and immune evasion in ovarian cancer [17]. Besides, emerging evidence suggests a potential regulatory relationship between BUB1B and the Wnt/*β*‐catenin pathway. In mice with a low expression mutation of BUB1B, developmental defects in the body and brain were effectively rescued by genetic inhibition of sFRP3, an endogenous Wnt antagonist [18]. This finding was further corroborated by another study showing that sFRP3 ameliorated neural inflammation caused by BUB1B mutation [19]. Additionally, BA et al. reported significant alterations in Wnt/*β*‐catenin signaling activity in BUB1B knockout hearts [20]. Consequently, BUB1B was hypothesized to facilitate the progression of ovarian cancer by modulating the Wnt/*β*‐catenin pathway in our study.

The present study revealed BUB1B as a hub gene showing differential expression between normal ovarian tissues and OC tissues. Therefore, this study investigated whether BUB1B participates in metastasis and its functional mechanism in OC. Our research provides evidence for elucidating the mechanisms underlying the progression of OC and offers a novel therapeutic target for OC treatment.

## 2 Materials and Methods

### 2.1 Bioinformatics Data Processing

The GSE18520 and GSE14407 datasets, containing 65 OC tumor tissue samples and 22 normal ovarian tissue samples, were obtained from the GEO database (https://www.ncbi.nlm.nih.gov/geo). The mRNA sequencing data for 379 OC samples were downloaded from the TCGA database (https://www.cancer.gov/about‐nci/organization/ccg/research/structural‐genomics/tcga), and 88 ovarian normal samples were downloaded from GTEx (https://commonfund.nih.gov/GTEx/). DEGs were analyzed by the limma package. The hub genes were calculated by Cytohubba, and BUB1B was selected for further research. Survival analysis of BUB1B was performed by using the KM‐PLOTTER database. The GEPIA website was utilized to evaluate the expression of BUB1B in OC and normal tissues (http://gepia.cancer‐pku.cn/). The Tumor Immune Estimation Resource (TIMER) website was employed to analyze the correlation between BUB1B expression and tumor‐infiltrating immune cells (https://cistrome.shinyapps.io/timer/).

To further explore the regulatory effects of BUB1B on the Wnt/*β*‐catenin pathway, the transcriptome data of OC tissues were grouped based on their BUB1B expression levels for differential gene expression (DGE) analysis. Additionally, gene set enrichment analysis (GSEA) was conducted using the clusterProfiler package to detect the changes in the Wnt/*β*‐catenin pathway in the BUB1B high‐expressing group.

### 2.2 Cell Culture

OC cell lines (SKOV‐3, HO‐8910, and A2780) and a normal ovarian epithelial cell line (IOSE80) were derived from EXPASY or ATCC and stored in liquid nitrogen. The cells were appropriately recovered and cultured in RPMI‐1640 medium (Life Technology) containing 10% fetal bovine serum (FBS, Gibco, Carlsbad, CA, USA), penicillin (100 units/mL), and streptomycin (100 μg/mL) at 37°C in a humidified atmosphere containing 5% CO_2_.

### 2.3 Quantitative Real‐Time PCR

RNA was extracted using TRIzol (Takara, Japan) and reverse transcribed into cDNA using the Prime Script RT‐PCR kit (Takara). Subsequently, quantitative real‐time PCR (qRT‐PCR) was performed using SYBR Premix Ex Taq (Takara) on the Applied Biosystems 7500 Real‐time PCR system. The relative expression of BUB1B (forward primer: CTGTATGCCTCTGGTCGTAC; reverse primer: TGATGTCACGCACGATTTCC) was evaluated by the 2^−ΔΔCt^ method, with *β*‐actin as the internal reference gene (forward primer: CTGTATGCCTCTGGTCGTAC; reverse primer: TGATGTCACGCACGATTTCC).

### 2.4 Western Blot

Total protein was extracted by 1 × sodium dodecyl sulfate (SDS) buffer with proteinase inhibitor, phosphatase inhibitor, and PMSF (phenyl methane sulfonyl fluoride). After being separated by SDS polyacrylamide gel electrophoresis (SDS‐PAGE), protein bands were transferred to a polyvinylidene difluoride membrane (Bio‐Rad, Hercules, CA, USA). Membranes were blocked with 5% skim milk for 1 h, followed by incubation with primary antibodies against BUB1B (Servicebio, China, 1:2000), *β*‐catenin (Servicebio, 1:1000), Cyclin D1 (Servicebio, 1:800), c‐myc (Servicebio, 1:1000), or *β*‐actin (Servicebio, 1:5000) at 4°C overnight. After washing, the membranes were further incubated with HRP‐conjugated secondary antibodies for 1.5 h. Finally, the target protein was visualized with the ECL chemiluminescence kit (Servicebio).

### 2.5 BUB1B Intervention Experiment

The shRNA vector for BUB1B knockdown (sh‐BUB1B) and negative control (sh‐NC), BUB1B overexpression plasmids (BUB1B) and negative control (vector) were purchased from Genepharma Co. Ltd. (Shanghai, China). The plasmids were transfected into SKOV‐3 and HO‐8910 cells using Lipofectamine 2000 (Invitrogen, Carlsbad, CA, USA) according to the manufacturer's instructions. Transfected cells were cultured in RPMI‐1640 medium supplemented with 2 μg/mL puromycin to select stable cells.

### 2.6 Cell Proliferation Detection

SKOV‐3 and HO‐8910 cells transfected with sh‐NC or sh‐BUB1B were seeded in 96‐well plates at a density of 2000 cells/well. Then, cell activity at 1, 2, 3, and 4 days after transfection was determined using a cell counting kit‐8 (CCK‐8, Dojindo Molecular Technologies, Kumamoto, Japan) assay. The reactions were incubated for 1 h at 37°C. The absorbance at a wavelength of 450 nm was measured using a Labsystem multiskan microplate reader (Merck Eurolab, Dietikon, Switzerland). For the 5‐Ethynyl‐2′‐deoxyuridine (EdU) assay, cells were seeded in 96‐well plates and incubated for 24 h. EdU (50 nmol/mL, Ribobio, China) was added for incubation for 2 h at 37°C. Then, cells were fixed with 4% polyformaldehyde for 20 min and permeabilized with 0.5% Triton X‐100 for 10 min. ApolloR reaction cocktail and Hoechst 33,342 were added sequentially. The images were captured under a microscope (Olympus, Japan), with red indicating EdU‐positive cells. Each group contained five duplicates and was independently performed in triplicate.

### 2.7 Wound Healing Assay

Cells in each group were planted into 6‐well plates and incubated for 1 day to 90% confluence. A 0.2‐mL pipette tip was utilized to make a straight scratch along the diameter of the well. Images of the wound were acquired at 0 and 24 h after scratching. The change in the width of the wound was measured to calculate the inhibitory rate of migration.

### 2.8 Apoptosis Detection

Apoptosis was analyzed by flow cytometry using an Annexin V‐FITC/PI apoptosis detection kit (Vazyme Biotech, Nanjing, China). Cells from each group were collected and resuspended in 0.5 mL binding buffer at a density of 1 × 10^5^ cells/mL. Then, the cells were stained with 5 μL Annexin V‐FITC and 5 μL PI for 10 min in the dark. Finally, cell apoptosis was detected using a FACSVerse flow cytometer (BD Biosciences, San Jose, CA, USA).

### 2.9 Transwell Assay

A Transwell assay was utilized to evaluate cell invasion. The upper chambers were coated with Matrigel (Corning, NY, USA). SKOV‐3 and HO‐8910 cells transfected with sh‐BUB1B or sh‐NC vectors were suspended in RPMI‐1640 medium without FBS (2*10^4^ cells/100 μL), and 200 μL of cells were seeded in the upper chamber. Moreover, 800 μL of RPMI‐1640 medium with 10% FBS was added to the lower chamber. After 24 h of incubation, invaded cells were fixed and stained with 0.1% (w/v) crystal violet solution. Images were captured and cells were counted in three random regions.

### 2.10 Tumor Xenograft Model

Twenty‐four BALB/c nude mice (four‐week‐old, female) were purchased from the Experimental Animal Center of Southern Medical University. The mice were randomly divided into four groups, including six mice in each group: SKOV3‐sh‐NC, SKOV3‐sh‐BUB1B, HO8910‐sh‐NC, and HO8910‐sh‐BUB1B. A suspension of 5 × 10^6^ cells in Matrigel (1:1 v/v) was injected into the armpits of nude mice to establish a transplanted tumor model. Tumors were measured every 3 days starting from day 10 after injection to calculate tumor volume. At day 31 (when the maximum long diameter of the tumor did not exceed 2 cm), all nude mice were euthanized under anesthesia and photographed. The tumors were dissected and photographed.

### 2.11 Immunohistochemistry Assay

Tissues were fixed with 4% formaldehyde, dehydrated using graded ethanol, embedded in paraffin, and cut into 4‐μm slices. The slices were loaded onto glass slides, then dewaxed and rehydrated. Next, the slices were blocked with 5% BSA and incubated with primary antibodies against Ki67 (1:100, Abcam, ab15580, USB) or caspase 3 (1:200, CST, #9661) at 4°C overnight. Then, the slices were incubated with secondary antibodies (Abcam, USA) at room temperature for 1 h. Subsequently, sections were dyed with 3,3′‐diaminobenzidine and hematoxylin, and images were obtained with a light microscope (Olympus).

### 2.12 Statistical Analysis

Statistical analyses were performed using GraphPad 7.0. The data are presented as the means ± SD. Differences between the two groups were estimated by two‐tailed Student's t‐tests, and one‐way ANOVA analysis was used for more than two groups. A *p*‐value of less than 0.05 was considered statistically significant.

## 3 Results

### 3.1 BUB1B Is Up‐Regulated in OC and Is Associated With Poor Survival

**FIGURE 1:**
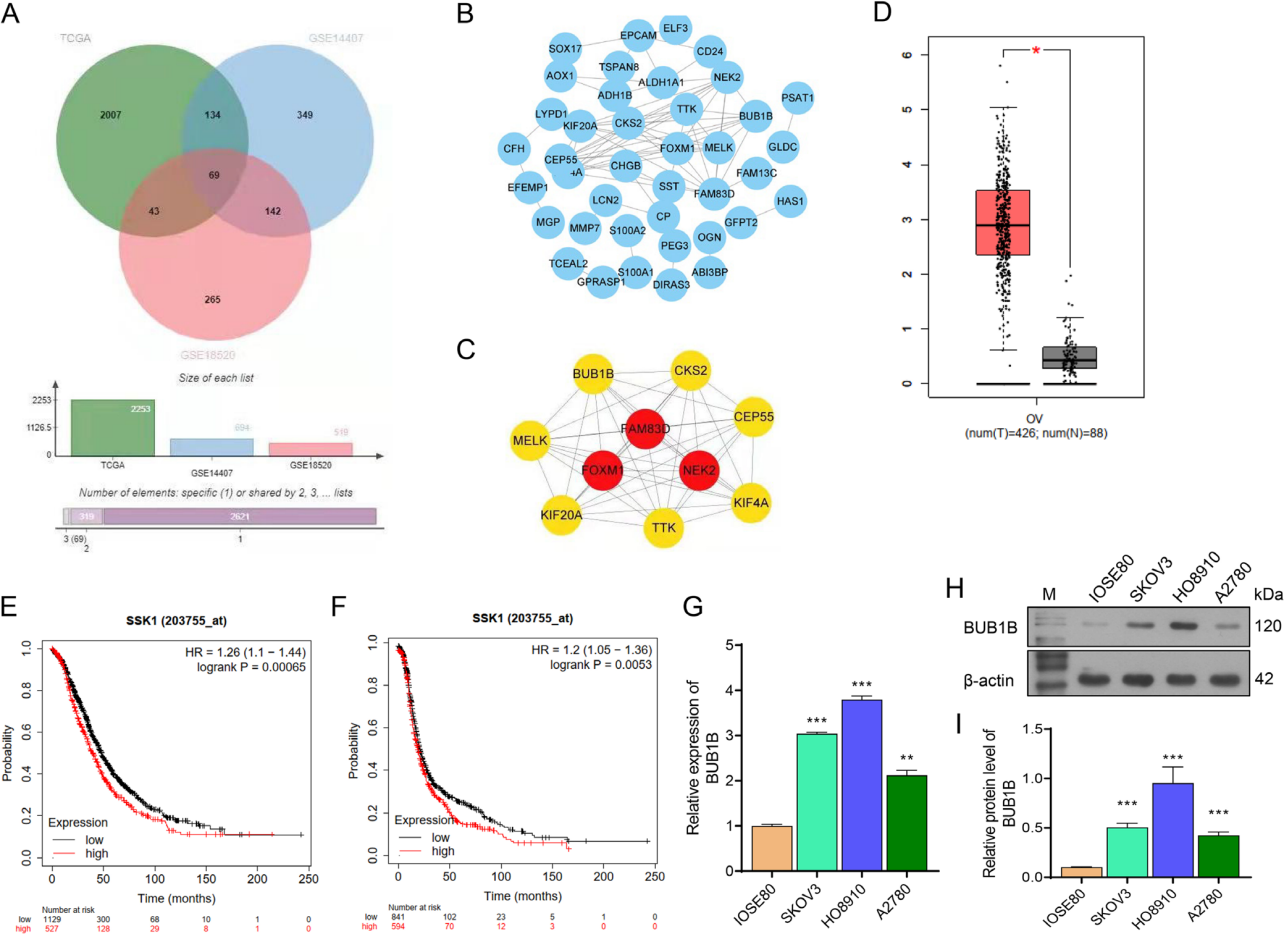
Bioinformatics analysis revealed the prognostic value of BUB1B in OC patients. (A) Venn diagram displaying the DEGs between OC tumor tissues and normal ovarian tissues in TCGA, GSE14407, and GSE18520 combined with GTEx. (B, C) Hub genes were calculated by Cytohubba. (D) BUB1B expression in OC tumor tissues and normal ovarian tissues. (E, F) The association between BUB1B expression and OS/PFS of OC patients. (G–I) BUB1B mRNA and protein expression in SKOV3, HO8910, A2780, and IOSE80 were detected by qRT‐PCR and western blot. *N* = 3. *p < 0.05, **p < 0.01, ***p < 0.001, one‐way ANOVA analysis.

Using the combined GTEx and TCGA databases, the DEGs between OC tumor tissues and normal ovarian tissues were analyzed, revealing 69 overlapping DEGs from GSE14407, GSE18520, and TCGA datasets (Figure 1A). Thereafter, the PPI protein network was constructed, and BUB1B was found to be a hub gene for further analyses (Figure 1B,C). The BUB1B expression was analyzed in GEPIA, showing that BUB1B expression in OC tissue was significantly higher than that in normal ovarian tissue (Figure 1D). Furthermore, the prognostic significance of BUB1B expression was evaluated using the KM‐PLOTTER database. The results revealed that OC patients with higher BUB1B expression had poor overall survival and progression‐free survival rates compared to those with lower BUB1B expression (Figure 1E,F). Furthermore, the BUB1B expression was significantly upregulated in OC cell lines (SKOV‐3, HO‐8910, and A2780) compared to that in the normal ovarian epithelial cell line IOSE80 (Figure 1G–I).

### 3.2 BUB1B Is Associated With Immune Cell Infiltration and the *β*‐Catenin Pathway

**FIGURE 2:**
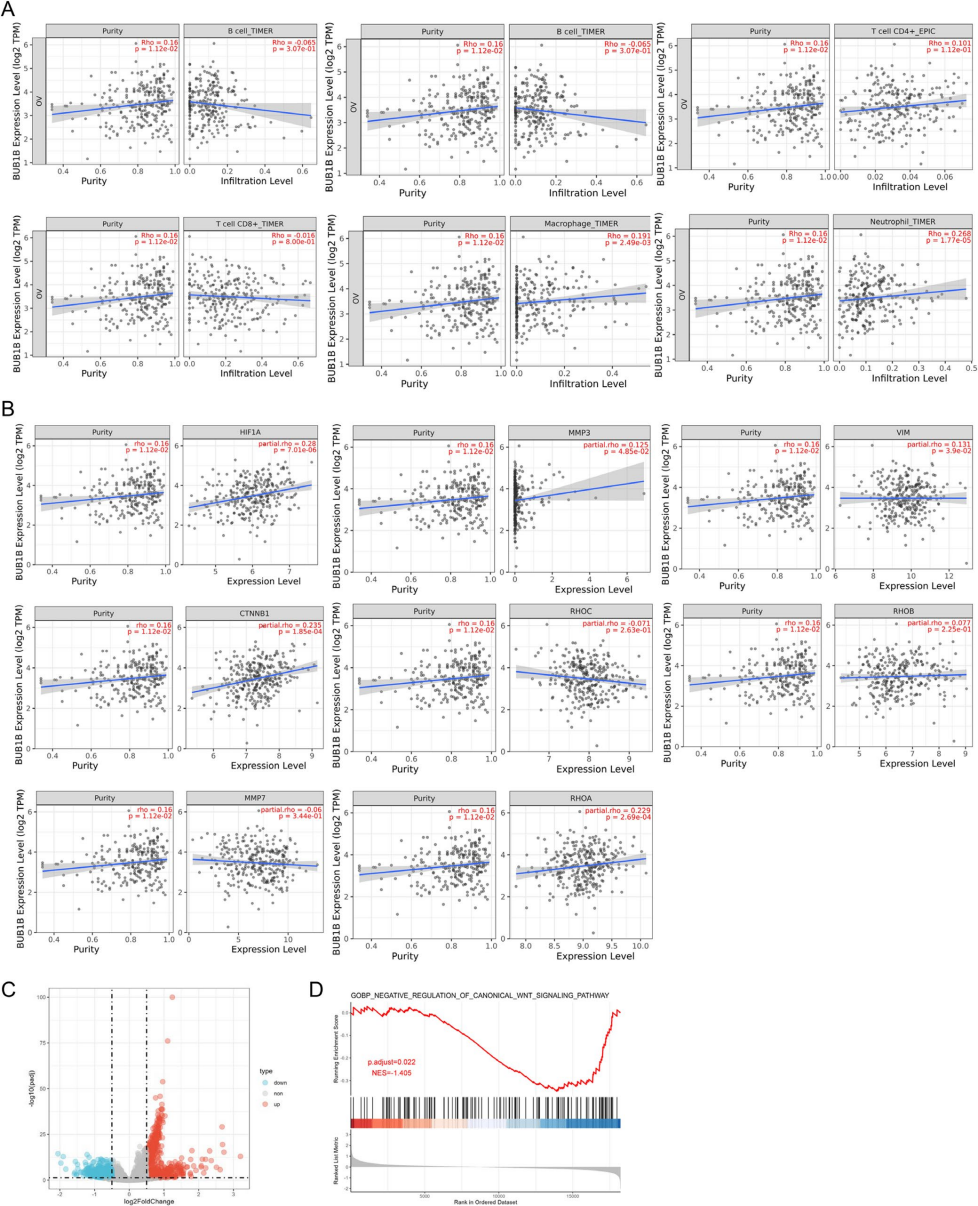
The correlation between BUB1B expression and immune cell infiltration or signaling pathways analyzed by the TIMER2.0 database. (A) The correlation between BUB1B expression and immune cell infiltration. (B) The correlation between BUB1B expression and the expression of HIF1A, MMP3, VIM, CTNNB1, RHOC, RHOB, MMP3, and RHOA in OC. (C) DGE analysis was performed on BUB1B‐expressing OC tissues from the TCGA database (logFC > 0.5, *p*.adjust < 0.05). (D) GSEA analysis was used to confirm the regulatory effects of BUB1B on the Wnt/*β*‐catenin pathway.

To explore the underlying function and mechanism of BUB1B in OC, the TIMER2.0 database was employed to reveal the relationship between BUB1B expression and infiltration of immune cells and signaling pathways in tumor tissues. The results revealed that a higher expression of BUB1B in OC tissues was associated with a higher proportion of tumor cells; moreover, infiltration of CD4 T+ cells, macrophages, and neutrophils also showed an increasing trend (Figure 2A). The findings also showed that the expression of BUB1B in OC tissues is positively related to HIF‐1α (HIF1A), MMP3, vimentin (VIM), *β*‐catenin (CTNNB1), and RhoA (RHOA) (Figure 2B), suggesting that BUB1B may be involved in these pathways. Previous research has reported the potential relationship between BUB1B and the Wnt/*β*‐catenin pathway [18]. Hence, the regulatory effects of BUB1B on the Wnt/*β*‐catenin pathway were analyzed using the TCGA database. OC tissues were grouped based on BUB1B expression, revealing 692 downregulated genes and 734 upregulated genes (Figure 2C). Notably, the negative regulation of the Wnt signaling pathway was alleviated in the high BUB1B expression group, indicating the activation of the Wnt/*β*‐catenin pathway (Figure 2D).

### 3.3 BUB1B Knockdown Inhibits Proliferation, Migration, and Invasion, and the Wnt/*β*‐Catenin Pathway in OC

**FIGURE 3:**
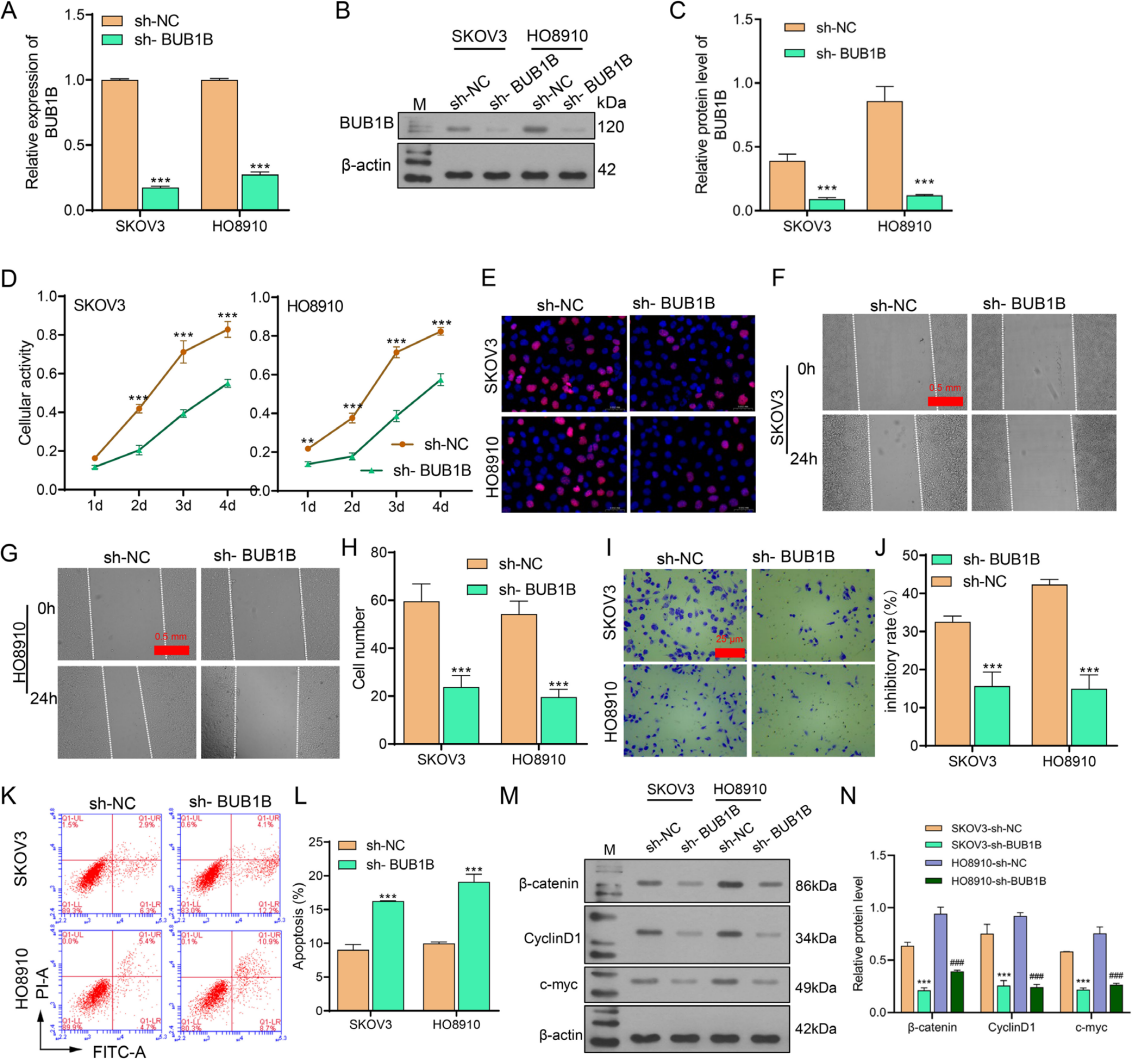
BUB1B knockdown inhibited the malignant behaviors of OC cells. (A–C) The efficiency of BUB1B knockdown in SKOV‐3 and HO8910 was detected by qRT‐PCR and western blot. (D) CCK‐8 assay was performed to detect cell activity on days 1, 2, 3, and 4 after BUB1B knockdown. (E) Cell proliferation capacity was assessed by Edu staining. (F–H) Cell migration was detected by wound healing assay. (I,J) Cell invasion was detected by the transwell assay. (K,L) The proportion of cell apoptosis was measured by flow cytometry. (M, N) The expression of *β*‐catenin, Cyclin D1, and c‐myc was detected by Western blot. *N* = 3. **p < 0.01, ***p < 0.001 vs. sh‐NC or SKOV3‐shNC; *p* < 0.001 vs. HO8910‐shNC, *t* tests. NC, negative control.

Next, the two OC cell lines with the highest BUB1B expression (SKOV3 and HO8910) were used for the BUB1B knockdown assay to demonstrate the effect of BUB1B on proliferation, migration, and invasion in OC cell lines. qRT‐PCR and western blot assays showed significantly lower BUB1B expression at both mRNA and protein levels in the sh‐BUB1B groups (Figure 3A–C). CCK‐8 and EdU assays revealed that BUB1B knockdown significantly inhibited OC cell proliferation (Figure 3D,E). Wound healing and transwell assays showed that OC cell migration and invasion were also significantly inhibited by BUB1B knockdown (Figure 3F–J). In addition, the flow cytometry assay demonstrated that cell apoptosis was significantly promoted by BUB1B knockdown (Figure 3K,L). Furthermore, the expression of proteins involved in the Wnt/*β*‐catenin signaling pathway was examined. The results showed that *β*‐catenin, CyclinD1, and c‐myc were downregulated when BUB1B expression was suppressed (Figure 3M,N).

### 3.4 BUB1B Regulates Malignant Behavior in OC via the Wnt/*β*‐Catenin Pathway

**FIGURE 4:**
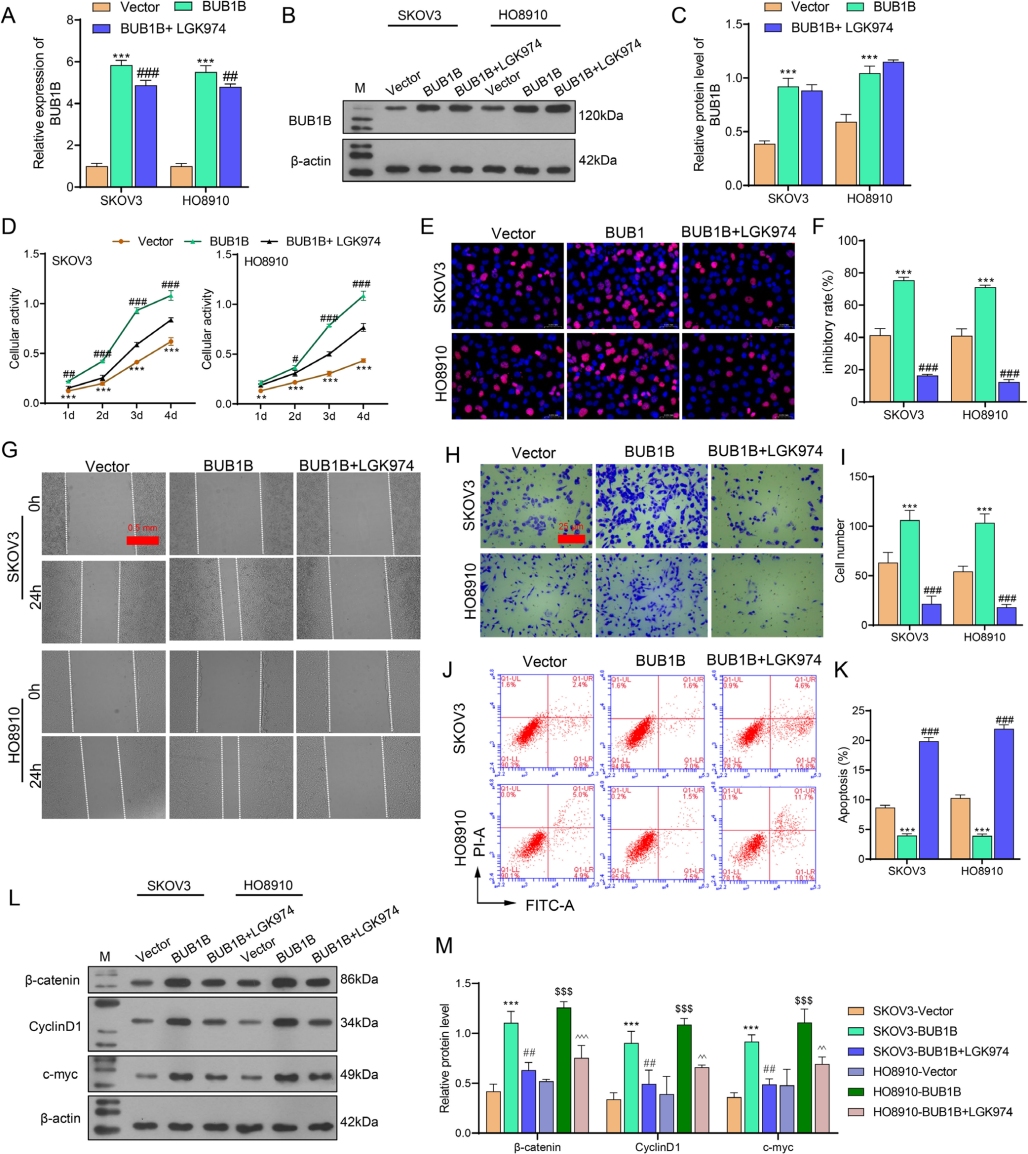
BUB1B promoted the malignant behaviors of OC cells by regulating the Wnt/*β*‐catenin signaling pathway. OC cells transfected with BUB1B overexpression plasmids (BUB1B) or control plasmids (Vector) were co‐treated with the Wnt signaling inhibitor LGK974 (BUB1B + LGK974, 200 μg/mL). (A–C) BUB1B mRNA and protein expression detected by qRT‐PCR and western blot. (D) Cell activity on days 1, 2, 3, and 4 after BUB1B knockdown detected by CCK‐8 assay. (E) Cell proliferation capacity assessed by Edu staining. (F, G) Cell migration detected by the wound healing assay. (H, I) Cell invasion detected by the transwell assay. (J, K) The proportion of cell apoptosis measured by flow cytometry. (L, M) The expression of *β*‐catenin, Cyclin D1, and c‐myc detected by western blot. *N* = 3. **p < 0.01, ***p < 0.001 vs. Vector or SKOV3‐Vector. *p* < 0.01, *p* < 0.001 vs. BUB1B or SKOV3‐BUB1B. *p* < 0.001 vs. HO8910‐Vector. ^^*p* < 0.01, ^^^p < 0.001 vs. HO8910‐BUB1B, one‐way ANOVA analysis.

To demonstrate whether the Wnt/*β*‐catenin signaling pathway mediated the function of BUB1B in OC, LGK974 (200 μg/mL), an inhibitor targeting the Wnt/*β*‐catenin pathway, was applied. qRT‐PCR and western blot assays revealed that LGK974 had no obvious effect on BUB1B expression at both mRNA and protein levels (Figure 4A–C). However, LGK974 suppressed the cell proliferation, migration, and invasion induced by BUB1B overexpression (Figure 4D–I), and it also reversed the anti‐apoptotic effect of BUB1B overexpression (Figure 4J,K). In addition, the expression of *β*‐catenin, CyclinD1, and c‐myc was downregulated by treating with LGK974 (Figure 4L,M).

### 3.5 BUB1B Knockdown Inhibits the Tumorigenesis of OC In Vivo

**FIGURE 5:**
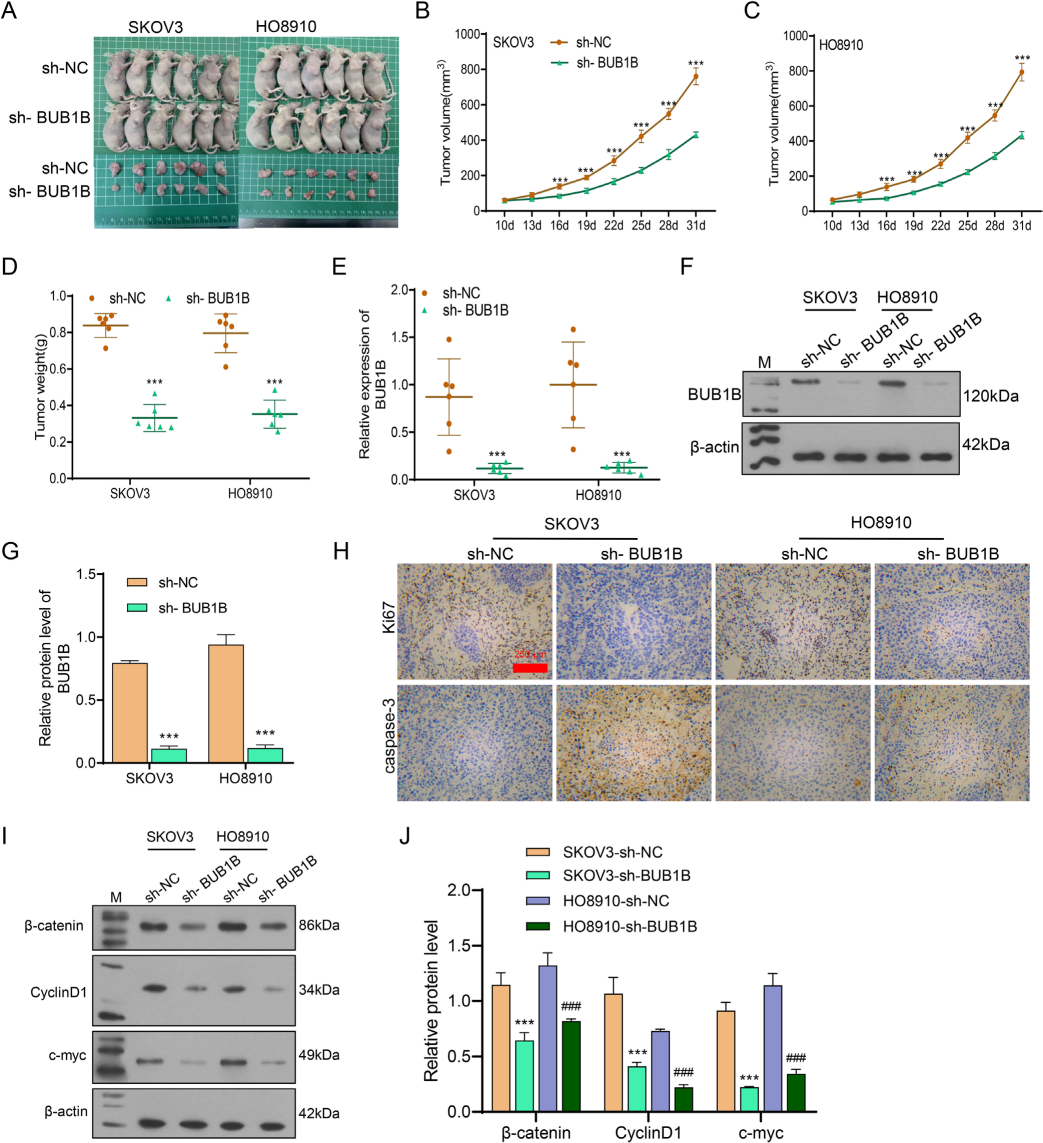
BUB1B knockdown inhibited tumor formation in vivo. OC cell lines transfected with BUB1B knockdown plasmids (sh‐BUB1B) or control plasmids (sh‐NC) were injected subcutaneously into the axillary region of nude mice to establish a xenograft tumor model (*n* = 6 per group). (A) The xenograft nude mice were sacrificed at day 31 after cell injection. (B–C) The growth curve of tumor volume was plotted based on measurements taken at days 10, 13, 16, 19, 22, 25, 28, and 31 after injection. (D) The weight of tumors from each group. (E–G) BUB1B mRNA and protein expression in the tumor tissues were detected by qRT‐PCR and Western blot. (H) Representative images of IHC for the expression of Ki67 and caspase‐3 in the tumor tissues. (I–J) The expression of *β*‐catenin, Cyclin D1, and c‐myc in the tumor tissues was detected by Western blot. *N* = 5. ***p < 0.001 vs. sh‐NC or SKOV3‐sh‐NC; *p* < 0.001 vs. HO8910‐sh‐NC, *t*‐tests.

To further elucidate the role of BUB1B in OC, an OC tumor xenograft model was established. At day 31 after injection, all the mice were killed, and the tumors were separated (Figure 5A). The weight and volume of tumors from sh‐BUB1B groups were significantly lower than those from sh‐NC groups (Figure 5B–D). qRT‐PCR and western blot assays revealed that BUB1B expression in tumor tissues was significantly reduced by BUB1B knockdown (Figure 5E–G). The IHC assay showed decreased Ki67 expression and increased caspase‐3 expression in sh‐BUB1B groups (Figure 5H), indicating that BUB1B knockdown promotes cell apoptosis. Moreover, the expression of *β*‐catenin, Cyclin D1, and c‐myc was downregulated when BUB1B expression was suppressed (Figure 5I,J).

## 4 Discussion

Understanding the mechanism of OC progression and metastasis is vital for OC treatment. To the best of our knowledge, this is the first report investigating the function and molecular mechanism of BUB1B in OC. This study confirmed that BUB1B expression was significantly upregulated in OC tissues and cell lines, and the high expression of BUB1B was associated with shorter OS and PFS in OC patients. BUB1B knockdown inhibited OC cell proliferation, migration, invasion, and tumor growth via the Wnt/*β*‐catenin signaling pathway, indicating the potential therapeutic value of BUB1B for OC.

As a unit of SAC, BUB1B plays an essential role in mitosis, effectively preventing aneuploidy, which is a common feature of cancer [21]. Abnormal BUB1B expression has been reported in cancer development, progression, immune resistance, chemoresistance, and radioresistance [11, 22, 23]. The present study revealed that BUB1B expression was significantly elevated in OC and negatively correlated with prognosis. In addition, BUB1B knockdown ameliorated the malignant behavior of OC and led to smaller OC tumor volume and weight in nude mice. Similarly, previous studies have reported abnormally high BUB1B expression in HCC, bladder cancer, prostate cancer, and other cancers. However, BUB1B showed opposite results in polyploid cells and colorectal adenocarcinomas. BUB1B induced polyploid cell apoptosis and inhibited the growth of tumors in nude mice with immune deficiency [24]. BUB1B expression was significantly lower in colorectal cancer, which was linked to poor prognosis and lymph node metastasis [25, 26]. Notably, the different roles of BUB1B in various types of cancer may be attributed to tumor heterogeneity. Although most studies have demonstrated the function of BUB1B in cancer without fully elucidating the underlying mechanisms, several functional mechanisms of BUB1B have still been reported. BUB1B evokes chromosomal instability via phosphorylation of CEP170 to promote myeloma malignancy [27]. In prostate cancer, BUB1B was found to accelerate cell proliferation by transcriptionally regulating MELK [28]. In addition, a few studies have reported that BUB1B expression is promoted by m6A modification by the m6A reader IGF2BP1 in NSCLC [23]. BUB1B expression is also transcriptionally manipulated by FOXM1, which activates the BUB1B promoter [22]. Collectively, the mechanisms underlying the effects of BUB1B in cancer remain incompletely understood. However, BUB1B represents a potential therapeutic target [29]. Considering the pivotal role of BUB1B, the upstream and downstream regulatory networks of BUB1B require further research.

To explore the function of BUB1B in OC, the TIMER database was utilized to analyze the relationship between BUB1B and the tumor immune microenvironment. The infiltration of CD4+ T cells, macrophages, and neutrophils was positively correlated with BUB1B expression (Figure 2A). BUB1B expression is positively related to the infiltration of CD4 + T cells and macrophages in papillary renal cell carcinoma [30]. In renal cell carcinoma, BUB1B expression is positively correlated with inflamed CD8+ T cells, nivolumab sensitivity, exhausted T‐cell signature, and IFN‐γ signature [31]. In endometrial cancer, BUB1B expression was found to be relevant to the infiltration of activated CD4 + T cells and CD8 + T cells [32]. Thus, BUB1B participates in reshaping the tumor immune environment. Nevertheless, the molecular mechanism linking BUB1B and the infiltration of immune cells has not yet been reported.

The Wnt/*β*‐catenin signaling pathway participates in many aspects of cellular regulation, such as cell proliferation, polarity, survival, and tissue homeostasis. This pathway is strictly regulated to ensure appropriate activity. However, multiple studies have reported abnormal Wnt/*β*‐catenin signaling pathway activity in the development of several pathologies, including cancers. A growing number of studies have found that the Wnt/*β*‐catenin signaling pathway plays a vital role in OC. For example, Ji et al. reported that Wnt/*β*‐catenin‐TCF4‐FOXP4‐PTK7 established a positive feedback loop to facilitate cell proliferation, migration, and EMT, promoting OC development [33]. FOXQ1 was found to facilitate OC progression by activating the Wnt/*β*‐catenin signaling pathway [34]. ANXA8 induces the activation of the Wnt/*β*‐catenin signaling pathway via UCHL5 to induce proliferation, invasion, and migration of OC cells [35]. In this study, the inhibitor of the Wnt/*β*‐catenin signaling pathway, LGK974, was used to inhibit OC cell proliferation, migration, and invasion induced by BUB1B overexpression (Figure 4).

The Wnt/*β*‐catenin signaling pathway mediates the effects of different cellular processes on cancer cell malignancies. Liu et al. revealed that Hexokinase 2, a rate‐limiting enzyme that catalyzes the phosphorylation of glucose in glucose metabolism, promotes cell proliferation and tumor growth through the Wnt/*β*‐catenin signaling pathway, further upregulating Cyclin D1 and c‐myc in epithelial OC [2]. The Wnt/*β*‐catenin signaling pathway responds to an imbalance in mitochondrial homeostasis. CBR‐5884, an inhibitor of PHGDH that disrupts mitochondrial redox homeostasis, increases the production of ROS and further inhibits the expression of Wnt/*β*‐catenin signaling pathway‐related indicators (such as *β*‐catenin, c‐myc, Cyclin D1, and Bcl2), thereby inhibiting OC progression [36]. Some contaminants, such as benzophenone‐1, promote abnormal proliferation and metastasis by activating the Wnt/*β*‐catenin signaling pathway [37]. In this study, based on the TIMER database, the expression of *β*‐catenin was positively correlated with BUB1B expression in OC (Figure 2B). In vitro and in vivo experiments showed that the activation of the Wnt/*β*‐catenin signaling pathway was regulated by BUB1B. Additionally, BUB1B knockdown decreased the expression of *β*‐catenin, Cyclin D1, and c‐myc (Figure 3), suggesting that BUB1B promotes OC metastasis via the Wnt/*β*‐catenin signaling pathway. In addition to metastasis, the Wnt/*β*‐catenin pathway also regulates various malignant behaviors of cancer cells. Song et al. reported that inactivation of Wnt/*β*‐catenin signaling by miR‐219‐5p increased OC cell sensitivity to cisplatin [38]. The *β*‐catenin signaling pathway regulates tumor angiogenesis. *β*‐catenin knockdown significantly interferes with angiogenic tube formation in human umbilical vein endothelial cells [39]. Moreover, the Wnt/*β*‐catenin signaling pathway is involved in cell ferroptosis and cuproptosis. The transcription complex formed from *β*‐catenin and TCF4 directly binds to the GPX4 promoter (a key regulator of ferroptosis) and enhances its expression, thereby suppressing ferroptosis [40]. In lung squamous cell cancer, activation of the Wnt/*β*‐catenin signaling pathway by FAM83A inhibits ferroptosis and promotes cell growth [35]. Wnt/*β*‐catenin blockade by specific inhibitors or TCF4 knockdown was found to sensitize cancer stem cells to elesclomol–Cuprum‐induced cuproptosis [41]. Collectively, the Wnt/*β*‐catenin signaling pathway was regulated by BUB1B, thereby further promoting OC metastasis. Our study provides a new sight for OC progression.

## 5 Conclusion

Our study confirmed that BUB1B was upregulated in OC tissues and cell lines, and this abnormally high BUB1B expression was associated with poor OS and PFS. BUB1B knockdown inhibits the proliferation, migration, invasion, and metastasis of OC in vitro and in vivo. Moreover, the Wnt/*β*‐catenin pathway was identified as a medium through which BUB1B functions in OC. It provided a novel therapeutic strategy for OC therapy.

## Author Contributions

Conceptualization: Jing Wang, Xiaoling Su, Nan Lin. Data curation and formal analysis: Jing Wang, Xiaoling Su. Investigation and methodology: Jing Wang, Xiaoling Su, Tao Su. Validation and visualization: Jing Wang, Xiaoling Su, Nan Lin, Tao Su. Writing original draft: Jing Wang. Writing review and editing: Nan Lin, Tao Su.

## Ethics Statement

The animal study protocol was approved by the Institutional Ethics Committee of International Peace Maternal and Child Health Hospital ([GKLW]2020–22).

## Consent

The authors have nothing to report.

## Conflicts of Interest

The authors declare no conflicts of interest.

## Data Availability

The data that support the findings of this study are available from the corresponding author upon reasonable request.
